# An Analysis of Welfare Standards Within Tiger (*Panthera tigris*) Facilities in Thailand

**DOI:** 10.1002/zoo.21866

**Published:** 2024-09-16

**Authors:** Tanya S. Erzinçlioğlu, Georgina Groves, Samantha Ward

**Affiliations:** ^1^ For Tigers Cambridge UK; ^2^ Wild Welfare London UK; ^3^ School of Animal, Rural and Environmental Science Nottingham Trent University Nottingham UK

**Keywords:** animal welfare, enclosure design, mental state, Thailand, tigers

## Abstract

In Thailand, tigers are more numerous in captivity than they are in the wild, with 51 facilities housing approximately 1962 tigers. As charismatic fauna, tigers are popular with tourists, and the majority of facilities offer a variety of entertainment activities with controversial reports towards the animals' welfare. The aim of this research was to investigate tiger welfare in Thai zoos to identify specific welfare issues. We assessed 34 tourism facilities holding tigers in Thailand in 2019 using a 25‐point welfare assessment that utilizes the Five Domain model (incorporating all five domains: nutrition, physical environment, health, behavioural interactions and mental state). The mental domain score was derived from the scores of the other four domains. Additional data were collected from each facility, including the number of tigers, any colour variants, types of human interaction and admission cost. Welfare scores for each domain were calculated by totalling the scores per domain and dividing by the number of points allocated to that domain. A multiple regression was used to identify any significant predictors of mental domain score. The results revealed that the provision of a suitable physical environment scored the lowest, while nutrition scored the highest though this was still a low score overall. The multiple regression showed that 45.4% of the variance for the mental domain score was significantly affected by the number of colour variants housed and the types of human interaction available with facilities, with more of both these factors contributing to a more negative score. Our results demonstrate the need for urgent, comprehensive infrastructural, species‐appropriate environment and design and animal management improvements to increase animal welfare. Informed institutional change toward the breeding and use of tigers for public interactions is also required. This is the first assessment completed of captive tiger welfare of tiger facilities in Thailand and shows that the welfare concerns encountered are within a high proportion (67%) of Thailand's facilities. This supports the need for the creation and enforcement of effective and clear captive wildlife operational standards to provide a sustained solution for captive tiger welfare and can provide a considered approach to ex‐situ tiger management that, in conjunction with in‐situ efforts, can improve much needed conservation efforts of this species.

## Summary

- Physical environment impacted welfare scores the most.
- Increased numbers of colour variants and entertainment level decreases mental state score.
- Priority to improve species‐appropriate environment and design for captive tigers in Thailand.

## Graphical abstract

Assessed facilities that housed white tigers and had higher incidences of tiger entertainment had lower welfare scores. Welfare scores were highest, whereas facilities had better scores for the tigers' physical environment.

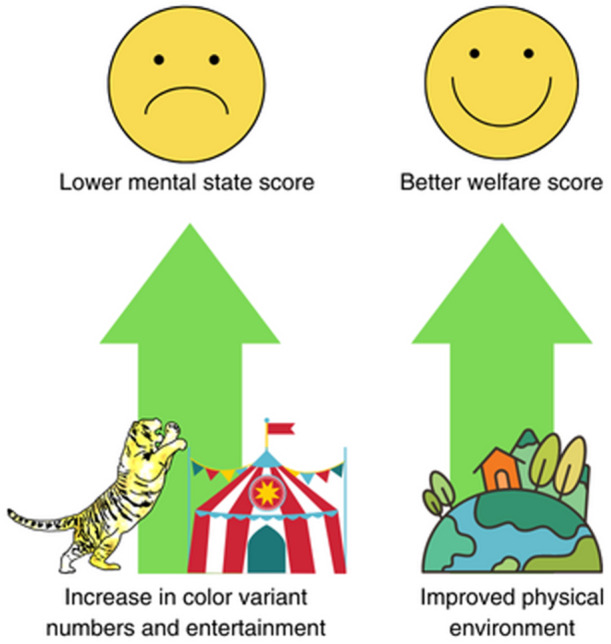

## 1 Introduction

Wild tiger (*Panthera tigris*) populations have been decimated over the last 100 years (Sanderson et al. 2010), due to extensive trophy hunting in the twentieth century, habitat loss, human–wildlife conflicts and an illegal trade fuelled by an insatiable demand for tiger parts (Environmental Investigation Agency [EIA] 2020). Best estimates indicate that only 4500 tigers remain in the wild across all of their range countries (World Wildlife Fund [WWF] 2022) globally. In contrast, captive populations are increasing, with an estimated 12,500 tigers in captivity worldwide (Convention on International Trade in Endangered Species of Wild Fauna and Flora [CITES] 2019). The increase in the captive population is not only associated with conservation efforts, but also with the exploitation of tigers for profit, with tiger farms supplying both the wildlife trade (Nyhus, Tilson, and Hutchins 2010) and tourist attractions, where tigers are in high demand (Cohen 2012). While this is an issue of global relevance, the scale of the problem is particularly evident in Asia, where countries such as China, Laos, Vietnam and Thailand are all home to large captive tiger populations: there are now more than 8000 tigers in captivity across these four countries (EIA 2020).

Tigers have been bred in captivity in Thailand for more than two decades (Schmidt‐Burbach, Ronfot, and Srisangiam 2015). As charismatic megafauna, tigers attract tourists to facilities due to their large size, ‘cute factor’, endangered status and high entertainment value for viewers (Carr 2016). It is estimated that although there are only 160 tigers left in the wild in Thailand (Wipatayotin 2020), there are an estimated 1960 tigers held in captivity within the country (EIA 2020). Most of these animals are housed in licensed facilities such as private venues, public zoos (‘place or premise where the collection of Wild Animal is for the purposes of recreation and education for the public and for scientific research and is also a breeding place of Wild Animal thereof’, Thai Law, 2014) and tiger farms (‘a facility that breeds tigers for commercial sale and trade of tiger parts such as tiger bone wine’, EIA 2020). In contrast, private facilities are operated by individuals or companies requiring licences, and the government‐run facilities are either under the Zoological Parks Organization Thailand (ZPOT) or the Department of National Parks, Wildlife and Plant Conservation (DNP).

Robust national legislation is important for effective animal welfare protection for captive wild animals to ensure a high standard of welfare within all captive facilities. Gray (2017) suggested that where a country lacks effective animal welfare legislation, it also lacks the ethical oversight of a zoo association. Thailand's legislation is lacking in this area; the Animal Cruelty Prevention and Welfare Act only came into force in 2014 and is one of the shortest animal welfare legislation acts in the world (Ghosh 2016). Vaguely worded, with unclear definitions for animal welfare and cruelty, the law is difficult to enforce, contributing to generally low welfare standards throughout the country (Dorloh 2017). The Act does not include any scientific literature or reference welfare frameworks such as the Five Domains. This framework would provide an important, and hugely helpful outline of good welfare that could be used within the context of enforcement. Without them, there are no standards to which welfare can be measured. Currently, the task of enforcing this Welfare Act falls to the conservation‐oriented DNP. While visits can occur when a welfare issue is raised, the Act does not appear to require any enforcement reporting or follow‐through inspections.

Tourist facilities within Thailand are known to offer a variety of entertainment activities such as circus‐style shows where the tigers perform behaviours to entertain, tiger photo opportunities and tiger cub feeding requiring the removal of cubs from their mothers at a young age (Cohen 2012, 2013). These forms of entertainment have created numerous welfare concerns (Cohen 2013; Schmidt‐Burbach, Ronfot, and Srisangiam 2015), including forcing the tiger into stressful interactive situations (World Animal Protection [WAP] 2010; Isoux 2016), restraining by a short leash or chain (Cohen 2012), controlling within confined spaces or by keeping them awake for interactions, something that is contrary to their natural behaviour of sleeping significant portions of the day (Szokalski, Litchfield, and Foster 2012), thus affecting their welfare.

Animal welfare has had multiple definitions, but generally, now it is recognized that welfare involves biological functioning, affective state and natural behaviour opportunities (Mellor 2016). It is now commonly considered to refer to the affective state of an animal and how it is feeling (Koene 2013; Veasey 2020; Mellor 2016). Mellor et al.'s (2020) Five Domains model considers how the aspects of an animal's physical environment, nutritional health care and behavioural interactions (with the environment, other animals and humans) impacts its overall mental well‐being. Good operational management practices always provide appropriate pro‐active health care and dietary consideration tailored to the needs of individual animals, and a physical and social environment where an animal is able to express normal and natural behaviours while minimizing fear, stress or frustration (AZA Tiger Species Survival Plan 2016).

Captive tigers in Thailand across all facilities are frequently housed in inappropriate living conditions such as small and barren enclosures without suitable furnishings or enrichment (Schmidt‐Burbach, Ronfot, and Srisangiam 2015). Facilities often engage in speed‐breeding practices where high volumes of cubs are born to fulfil tourist demand for cub feeding and selfies (WAP 2016; Cohen 2012; Schmidt‐Burbach, Ronfot, and Srisangiam 2015). This means there are a high number of tigers per facility that result in overcrowding. Overcrowding typically results in a reduction in the quality of species‐specific enclosures, little to no enrichment and consequently the inability for tigers to perform natural behaviours (Schmidt‐Burbach, Ronfot, and Srisangiam 2015). Restrictions in performing natural behaviour are known to negatively affect both physical and mental health (Morgan and Tromborg 2007). Tigers are known to be solitary in nature (Szokalski, Litchfield, and Foster 2012), and overcrowded conditions can induce stress (Morgan and Tromborg 2007), reduce access to resources (Olsson, Wurbel, and Mench 2018), reduce daily operational management and limit proactive and reactive health care to each animal.

There has been little comparative research investigating animal welfare within zoos and wildlife venues in Thailand, specifically on tigers, an aspect needed to better understand the welfare concerns prevalent in facilities across the country. Therefore, the aim of this research was to investigate tiger welfare across 34 tiger facilities open to the public in Thailand in 2019. We compared the welfare assessment results across facilities to identify the most concerning welfare issues within the tourism venues.

## 2 Methods

### 2.1 Facilities

Thirty‐four of a possible 63 public entertainment facilities housing tigers in Thailand (EIA 2020) were selected for assessment in 2019 as these were the ones open to the public. Many of the 64 facilities are privately owned and do not allow visitors. Facilities included private venues, public zoos and government‐run sites. However, all are included under licensed entertainment facilities. Facilities were identified through the use of previously published reports (WAP 2016; EIA 2017). Facilities were located throughout Thailand, predominantly in the main tourist destinations: Pattaya, Phuket, Koh Samui, Hua Hin and Chiang Mai. All facilities were open to tourists and were visited during opening hours. Researchers visited the facilities anonymously without the knowledge of facilities (Corrigan, Ng, and Williamson 2010; Schmidt‐Burbach, Ronfot, and Srisangiam 2015), assessing what was visible to the general tourist and did not assess off‐limit areas. For this reason, individual facilities were allocated a random number once assessed to ensure anonymity.

### 2.2 Data Collection

A tiger welfare assessment tool consisting of 25 questions was designed based on the principles of the Five Domains model (Mellor et al. 2020) and on similar assessment protocols utilized by the charity, Wild Welfare (Ward et al. 2020). This included 24 factors relating to nutrition (domain 1), the physical environment (domain 2), health (domain 3), behavioural interactions (with the environment, other animals and humans: domain 4) and an additional factor on entertainment level (animals used in displays or as other commercial entities = 0, visitor–tiger interactions = 1, no interactions or displays = 2) (Supporting Information S1: Appendix 1). Factors were grouped as per their relevance to the four domains and scored with a 0, 1 or 2 (Table 1), that is, the higher score was indicative of a better provision of the resource in question. Each factor was provided a qualitative standard for the researcher to qualify the result against. While score 2 was the highest score, it is not necessarily indicative of optimal best practice standards, but rather considered known species requirements within the context provided. Guidelines to this scoring criteria can be found in Supporting Information S2: Appendix 2.

**Table 1:** Breakdown of factors and their scores (a full description of each factor can be found in Supporting Information S2: Appendix 2).

Domain	Factors	No. of facilities scored			Poor	Acceptable	Good
		0	1	2	%	%	%
1: Nutrition	Water provision	10	2	22	29	6	65
	Clean water	12	2	20	35	6	59
	Species‐appropriate diet	0	31	3	0	91	9
2: Physical environment	Enclosure size	21	10	3	62	29	9
	Species‐specific enclosure	13	15	6	38	44	18
	Pond access	16	15	3	47	44	9
	Shelter access	16	12	6	47	35	18
	Cleanliness	1	13	20	3	38	59
	Substrate variation	11	18	5	32	53	15
	Environmental noise	16	9	9	47	26	26
	Management knowledge	18	16	0	53	47	0
3: Health	Signs of inbreeding	22	0	12	65	0	35
	Signs of injury	6	6	22	18	18	65
	Signs of pain	15	3	16	44	9	47
	Body condition score	7	17	10	21	50	29
	Healthcare provided	4	17	13	12	50	38
4: Behaviour interactions	Signs of stereotypy	17	14	3	50	41	9
	Response to non‐threatening humans	2	22	10	6	65	29
	Positive treatment by staff	8	19	7	24	56	21
	Signs of human‐applied injury	11	0	23	32	0	68
	Positive behaviour observation	14	16	4	41	47	12
	Staff used physical force	8	1	25	24	3	74
	Enrichment provision	23	10	1	68	29	3
	Appropriate social grouping	6	11	17	18	32	50
5: Mental state	Colour variants observed	—	—	—	53	0	47
	Entertainment level	4	16	14	12	47	41

Scores were allocated per facility (not per individual animal) to get an overview of the tigers and, to ensure consistency and to capture critical welfare issues, scores were based on the worst‐case observed. For example, at one facility, there might be three tigers in an enclosure, four tigers in a concrete cage and two tigers chained. Scoring only the highest welfare would not give an accurate result, nor would it be reflective of the current welfare issues present in these facilities. Additionally, this methodology was used because if a facility has poor welfare scores, we assumed that most/all tigers within that facility will have the same poor welfare though this is a limitation of the study as not all individual tigers may have a negative score. That being said, facilities are typically homogenous, particularly with their enclosure infrastructure and management, thus individuals are likely to have similar care and husbandry conditions throughout a facility. As this was a snapshot assessment and situational analysis, the scores were based on the lowest observed situation to fully understand what areas need to be improved within facilities. It is for this reason that locations have not been disclosed. Because of the nature of data collection, it was not possible for individuals to be assessed, plus some tigers were unseen. Facility scores for each of the 25 questions were totalled to get a final score out of 50 (Table 2). Factors in the Behavioural Interactions domain (signs of stereotypy, positive treatment by staff, positive behaviour, staff used physical force) and body condition observations were performed using rapid assessment protocols, over 5‐min periods (Altmann 1974). All other observations were taken during the duration of the visit, which meant time of day was a limiting factor, particularly for diet, cleanliness, enrichment, behaviours where scores may have differed depending on the time of the visit. Any behavioural observations that considered the tiger's response to non‐threatening humans were recorded only after the rest of the assessment was carried out to avoid the researcher influencing this behavioural response through the tiger's initial reaction response. It is likely that there is a relationship between how tigers respond to unfamiliar, non‐threatening humans and the interaction types the tigers engage in, which would impact these interaction observations. Two researchers conducted the 25‐question assessments, and an interobserver reliability assessment of the same tigers at the same facility was conducted to ensure observations and scores were > 90% accurate.

**Table 2:** Total scores for each facility out of 50 where a score of 25 or less is considered poor welfare, 26–40 was acceptable and a score of 41 or above good welfare.

	Total score across all factors									
	1–5	6–10	11–15	16–20	21–25	26–30	31–35	36–40	41–45	46–50
No. of facilities	—	2	4	10	5	4	3	2	4	—

Additional data was collected through direct observations and brief unstructured questions with animal care (keeping) staff. These data included the total number of tigers at the facility (staff discussion), interaction type, the number of colour variants housed and how much it costs for general adult admission to the facility. These additional data were included as potential welfare indicators. The number of tigers was recorded as it could have a bearing on welfare, with a greater number of tigers requiring more resources, spreading them thin, subsequently resulting in poorer welfare conditions within the facility as a whole. Interaction types were included as tigers participating in more hands‐on or intense interactions could be experiencing higher levels of stress, potentially negatively impacting their mental state. Colour variants (recorded as a number of tigers observed) were included as they are recognized as genetically inbred and suggest a lack of management welfare knowledge. Additionally, numerous health problems are associated with tiger colour variants, specifically white tigers. The cost of admission was included as it might indicate whether the funds are available to invest in animal welfare.

Age and sex of the tigers were not noted due to possible inaccuracies when observing from a distance or when observing large groups. There was no weighting for any of the scored factors as they were deemed equally important.

### 2.3 Data Analysis

A proportion for each of the four domains was calculated by totalling the scores per domain and dividing by the number of questions allocated to that domain on a facility level. The combined proportions from each of the four domains were then used to infer the overall affective state in the fifth domain, mental health for the facility as a whole.

A multiple regression in SPSS Version 26 was used to investigate any influencing factors that may impact the mental domain score. Variables entered into the model were interaction types, the number of colour variants at the facility, the proportion of tigers assessed at the facility and the cost of admission.

### 2.4 Results

From the 34 facilities, there were 799 tigers that were observed and assessed as part of this welfare assessment that took into account the worst‐case scenarios. This represents 64% of the total number of tigers that were housed across the total number of facilities in Thailand.

**Figure 1:**
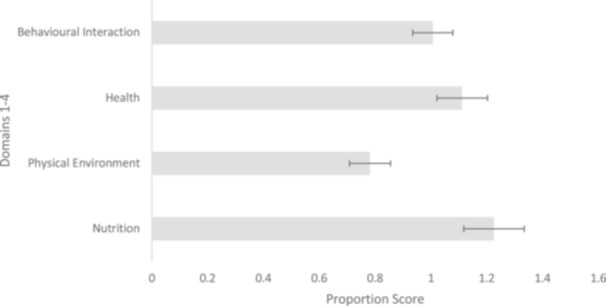
Mean proportion scores (±S.E bars) of the four domains, including nutrition, physical environment, health and behavioural interaction across 34 tiger facilities in Thailand.

Figure 1 shows that the lowest ratio score achieved across the 34 facilities was the physical environment provided for the tigers (mean 0.78 ± 0.07), the highest score was nutrition provision (mean 1.23 ± 0.11). The maximum score across each of the domains was 2.0, therefore, none of the domains scored particularly high across the facilities. Table 2 shows that most of the facilities offered suboptimal welfare, 21 facilities (62%) scored an overall score of 25 or lower, indicating ‘poor’ welfare, with only six facilities (18%) scoring above 35 for ‘good’ welfare practices. A total of 572 (72%) of the tigers assessed were found in the 21 facilities scoring five or less. In contrast to this, the six facilities that received a score of 36 or above collectively held 47 (36 observed) of Thailand's reported captive tigers. A total of 93 (12%) of observed tigers were one of three colour variants – white, snow or golden.

Nutrition scored well across most facilities with 65% providing water in some form. However, this reduced to 59% for clean water specifically. Ninety‐one percent of facilities provided an appropriate diet where raw food was provided but there was little to no variation within the diet. No facilities scored 0.

Physical Environment was poor across all factors graded. The majority of facilities (62%) had small living spaces, usually indicating no outdoor enclosure access and simple cage living. Only 18% of facilities had species‐specific enclosure designs, with just 9% of these providing a deep pond for swimming and an appropriate enclosure size, 18% providing shelter for all tigers, 15% a variety of substrates and 26% a quiet environment. In contrast, most facilities did not provide a tiger‐specific environment with no pond (47%), no shelter (47%), high levels of environmental noise (47%) and concrete substrate only (32%). Despite most facilities not achieving high scores for Physical Environment, the majority, 59%, scored good for cleanliness. None scored ‘good’ for management knowledge.

Under Health, 65% of facilities showed tigers with no sign of any injury. However, 44% of facilities had tigers showing signs of pain and 65% had signs of inbreeding, of which 53% had tiger colour variants, including white, snow and golden. Exactly half of the facilities had an average body condition score (BCS) indicating tigers were slightly over or under the ideal body condition. Twenty‐one percent of the facilities held tigers with poor BCSs (predominantly overweight).

In Behavioural Interactions, high levels of abnormal behaviours were seen in 50% of the facilities. No tigers were observed performing positive behaviours in 41% of facilities, with only 12% of the facilities had the majority of their tigers observed performing positive behaviours such as scent marking, playing or positive social interactions. Tigers were generally ambivalent in their response to unthreatening humans, with 65% of facilities having tigers respond in a neutral manner and only 6% (2 facilities) with tigers that responded very negatively, that is, moving away or acting in a fearful or aggressive manner (Supporting Information S2: Appendix 2). These results correlate with positive treatment by staff where 56% of facilities had hands‐on interactions but no force was observed, and 24% had interactions deemed rough or forceful. Human‐applied injury was noted in 32% of facilities with tigers declawed. The provision of enrichment was lacking or insufficient in 97% of the facilities within Thailand. Sixty‐eight percent of the facilities assessed scored 0, indicating that there was no enrichment at all, with only 3% providing an adequate enrichment infrastructure. Good social grouping was observed in 50% of facilities, with 18% having very poor grouping (two or more tigers kept in an area not appropriate for the number of tigers).

A multiple regression was used to predict the mental domain score from entertainment level, number of colour variant tigers, the proportion of tigers assessed and the admission cost. These variables significantly predicted 45.4% of the variance for the mental domain score (*F*_4,28_ = 4.985, *p* < 0.01). The number of colour variants and the entertainment level were found to significantly predict the mental domain score (*p* < 0.05). However, the admission cost and the proportion of tigers assessed were not significantly contributing to the model. The model suggested that as the number of colour variant tigers housed decreased and the entertainment level score increased (there was a reduction in entertainment activities, Supporting Information S2: Appendix 2), the mental domain score increased. The cost of entrance to the facility or the proportion of tigers assessed did not impact the mental domain score.

## 3 Discussion

The welfare assessment tool designed for this study was specifically to assess the needs and provisions for tigers based on the Five Domains (Mellor et al. 2020). The results showed that of the four domains the largest contributing factor to poor welfare in the assessed tiger facilities in Thailand were the physical environment, followed by behavioural interaction and health.

### 3.1 Nutrition

Despite nutrition scoring the highest, this does not mean that it was good across all facilities. Nutrition was the highest scoring, possibly as the provision of food and water are basic needs and this is in accordance with the Thai Animal Welfare Act's (2014) requirement of providing ‘sufficient habitation, food and water’. It is also possible that this domain scored higher due to most facilities providing palatable and sufficient water. However, while the majority of facilities provide water, some still do not do so sufficiently. There were still 10 facilities where no water was provided, leaving tigers panting and thirsty. Additionally, even when there was provision of water, the water quality was often lacking across facilities with algae present in water bowls. Ideally, tigers should have continued access to fresh, running water (AZA Tiger Species Survival Plan 2016).

While the quantity of food appeared to be adequate for most facilities, the study was unable to analyze more in‐depth diet provisions, such as the use of carcass feeding. Diet was accessed by determining whether the type of food provided was appropriate. Predominantly, raw chicken or various cuts of unknown meat were fed to tigers. Tigers should be given a raw diet with carnivore supplements where necessary (Dierenfeld et al. 1994). Additionally, the way the tigers were fed was assessed, with facilities scoring higher if they provided variation or food enrichment to encourage more natural hunting or foraging behaviours. There was little to no variation in the way the food was provided, with most facilities observed providing it on the floor or in bowls. A few facilities provided beef or pork once a week, with some using it as part of an enrichment programme, as observed and noted by the researchers. The volume of food provided per tiger was taken into consideration. For a healthy adult tiger, 4 kg of chicken per day is recommended (Dierenfeld et al. 1994) though researchers were unable to accurately assess this, it was inferred through body condition scoring as to whether tigers were fed appropriate amounts. It became evident that many facilities were likely overfeeding their tigers, particularly those that were interacting directly with the public.

### 3.2 Physical Environment

In the wild, tigers traverse anywhere from 7 to 60 km per day in search of food (Sunquist and Sunquist 2002), and there is a correlation between enclosure size and the distance paced by captive tigers (Breton and Barrot 2014). The majority of facilities in Thailand provide very small living spaces (typically 4 × 4 m) that do not provide the space for the environmental infrastructure that allows for such behaviours as running or climbing opportunities. Tigers were unable to perform exploratory, locomotory or patrolling behaviours, possibly resulting in a lack of mental and physical stimulation, and often, the emergence of negative behaviours such as abnormal repetitive behaviours (ARBs) arose from an inability to cope (Rose, Nash, and Riley 2017). Additionally, the small living spaces are predominantly barren, concrete‐floored cages – a third of all facilities only had concrete substrates. A range of substrates, including grass, sand and rocks should be provided throughout the living space to prevent health issues such as joint, skin or paw problems due to continuously living on concrete (AZA Tiger Species Survival Plan 2016; Croney et al. 2015).

Species‐appropriate enclosures should have den areas allowing respite from the public and conspecifics (if housed together) and promote resting or solitary behaviour requirements (AZA Tiger Species Survival Plan 2016; Lyons, Young, and Deag 1997). The researchers noted that retreat or visibility barriers were not provided within any facility, exposing the tigers to conditions where they lack control over their environmental use, which can contribute to feelings of stress and frustration.

A very small number of facilities did provide more enclosure furniture, such as platforms and dens, and a larger size where tigers were capable of performing running and climbing behaviours. Only six of the facilities (zoos rather than hands‐on tourist facilities) had living spaces meeting the American Association of Zoo (AZA) standards which are a minimum of 144 m^2^ per single tiger (AZA Tiger Species Survival Plan 2016). Importantly, AZA‐accredited facilities are encouraged to exceed this minimum and the average exhibit size is 510 m^2^, which none of the Thai facilities we assessed achieved. Additionally, enclosures require multiple elevated platforms (AZA Tiger Species Survival Plan 2016; Lyons, Young, and Deag 1997) and access to a pond as cage furnishings. Due to Thailand's hot climate, tigers should be provided with a pond to aid in thermoregulation (Yang, Fingean, and Brown 2013; Stryker et al. 2019) as well as to encourage a wider behavioural repertoire (Veasey 2020) and a potential reduction in ARBs (Biolatti et al. 2015). Despite this, almost half of the facilities did not provide pools or a submergible water source for the tigers.

It is unknown how often tigers had access to the outdoor enclosures. It is common practice for zoos to hold animals in indoor enclosure spaces at night for safety precautions (The Scottish Government 2019), and rotate individuals when there is a lack of space or incompatibility within social groups, resulting in animals being held in often, even smaller enclosure spaces, or even if all tigers had access to these areas. However, given the number of indoor cages with access to each enclosure, and the number of tigers observed in each enclosure at a given time, it is likely that many of the tigers do not get the opportunity to spend a significant portion of the 24‐h day in the outside space. In many facilities, it was common to see four or more indoor cages leading out into the same, single outside enclosure with only one or two tigers outside and other tigers viewable in the indoor areas. All facilities were only visited during public opening hours. Researchers did not visit the night‐time living areas and thus assessment of which would have provided a more comprehensive welfare analysis.

Two‐thirds of the facilities maintained high standards of cleanliness. This included the lack of faeces or litter within any living spaces. One‐third of the facilities had low standards of cleanliness; scum or green water in or around water bowls, faeces in piles usually near the entrance to the enclosure and some larger enclosures contained debris such as rusty fences. Clean water is important because all cats are sensitive to smells and will often not drink contaminated water (AZA Tiger Species Survival Plan 2016), possibly leading to dehydration. The removal of faeces is important because they can transmit or contain endoparasites (AZA Tiger Species Survival Plan 2016).

Impressions are generally more favourable when visitors visit somewhere clean and natural as the supposition is that the animals are cared for as well (Melfi, McCormick, and Gibbs 2004; Godinez and Fernandez 2019; Reade and Waran 1996). Arguably, these cleaner living spaces can promote better welfare, but overcleaning can also contribute to a sterile, void of choice and challenges as the facility places cleanliness above enrichment factors. As tigers scent mark, thorough disinfecting should be done rarely. It is also advised to leave some traces of habitation or faeces markings undisturbed (AZA Tiger Species Survival Plan 2016).

### 3.3 Health

The overall health of the tigers had a significant effect on welfare scores. Injury, BCS and disease impacted captive tiger welfare. Injuries such as wounds from fighting, sores from concrete (i.e., inappropriate housing), as well as neck injuries from chains, missing tails and swellings were observed. As both old and new injuries were recorded, it was indicative that injuries and body condition remain an ongoing issue. Almost a third of the facilities in 2019 were observed to have declawed tigers. Performed for safety purposes during interactions, declawing can cause severe injury, pain and deformities, especially with the onset of old age (Clark et al. 2014), subsequently negatively affecting mental and behavioural states.

Pain may occur in conjunction with injury. Signs of pain included limping, diarrhoea, hunching, stiff movement and coughing or wheezing (often a sign of a more serious medical condition) (Supporting Information S1: Appendix 1), though these are only the most basic of observations. Without consistent and thorough observation with experienced keepers and vets, it is possible that signs of pain went unnoticed. This may explain why the pain scores were at both extremes (Table 1). The fact that such a high rate of pain was observed, given the nature of tigers to hide pain, is in itself concerning and indicative of severe welfare issues.

Tigers in 39% of the facilities were considered to have good BCS, where feline bodies were lean, muscular with definition across the hindquarters, abdomen and shoulders (Fazio 2020). While, half of the observed tigers scored either side of this ideal (slightly over or underweight), almost one quarter (21%) had a poor BCS. The main reason for poor BCS was because the tigers were overweight – clear fat deposits and no definition across the hindquarters, abdomen or shoulders (Fazio 2020) rather than malnourished. Captive big cats are often overweight due to a lack of proper physical exercise as well as an unbalanced diet (Dierenfeld et al. 1994) which links back to the nutritional issues previously mentioned. The prevalence of obesity raises concerns regarding the monitoring of food intake, the quality of food provided and the inability to exercise. Obese tigers may suffer a multitude of related health problems, including reduced mobility, diabetes, liver problems, arthritis, respiratory issues and more (Tilson and Seal 1987). The lack of enrichment programmes that requires movement or the need for appetitive foraging contributes to the problem of obesity in many species of captive wildlife (Dierenfeld et al. 1994: Mishra et al. 2021). Additionally, some facilities may intentionally overfeed their tigers who interact with tourists to suppress the tiger's appetite to increase lethargy, which makes these tigers easier to handle during human–animal interactions (Pers. Obs., 2010–2020).

### 3.4 Behavioural Interactions

We considered the lack of appropriate environmental enrichment such as logs, hanging toys, balls, tyres and food enrichment as having a negative impact on welfare (see Supporting Information S2: Appendix 2). While some facilities did provide enrichment, it was limited to hanging tires, ropes around trees and balls. Two‐thirds of the facilities had no enrichment whatsoever, indicating a fundamental welfare issue. The provision of appropriate environmental enrichment is important in captivity for the promotion of highly motivated and rewarding behaviours that consequently elicit positive welfare states through a more complex environment (Skibiel, Trevino, and Naugher 2007). The lack of species‐appropriate enrichment and environments can lead to frustration and stress, and ARBs (Damasceno et al. 2017) such as pacing (fixed, repetitive walking along the same path without an apparent goal with a minimum of two repetitions; McPhee 2002) or overgrooming, both of which were observed at various levels across most facilities. Species‐appropriate enrichment alleviates boredom, encourages highly motivated behaviours and provides the tiger with challenges and choices which facilitate a degree of control within their environment (Carlstead and Shepherdson 1994; Ritzler et al. 2021). To prevent desensitization to enrichment items and to stimulate the tiger, enrichment methods should be varied (Skibiel, Trevino, and Naugher 2007; Szokalski, Litchfield, and Foster 2012; Tarou and Bashaw 2007) and include a wide range of habitats, sensory, nutritional, social and cognitive enrichment something lacking in these facilities with only one offering a well‐rounded enrichment programme. Each of these enrichment types can optimize tiger welfare in different ways with some overlap in providing optimum tiger welfare.

Many facilities in Thailand keep large numbers of tigers in close quarters, leading to welfare concerns regarding inappropriate social grouping. Results showed a wide variation regarding social grouping, with 50% deemed to have good social grouping and 19% with poor where two or more tigers were kept in living spaces not large enough to provide space and resources for all tigers. This score was based on international tiger housing guidelines (AZA Tiger Species Survival Plan 2016) and space constraints in that it should be 144 m^2^ for a single tiger, an added 50% for each additional tiger and that tigers should be kept with just two or three conspecifics (AZA Tiger Species Survival Plan 2016). Wild tigers are known to be solitary in nature, and the keeping of large numbers of tigers in close proximity to each other, could have a chronic effect on welfare (Szokalski, Litchfield, and Foster 2012). Too many tigers in a small space result in an inability to access resources or to retreat from conspecifics, promoting frustrated or agonistic behaviours which can result in ARBs or injuries (Galardi et al. 2021). However, even in the wild, tiger social structure may not be as simple with nonaggressive interactions documented, indicating it is possible that tigers may be more social than current literature suggests (Galardi et al. 2021). In fact, tigers raised together in captivity often display strong play, social and other behaviours with suitable conspecifics though such captive tiger social interactions could be the result of habituation rather than an innate need to interact given the captive tiger environment is so removed from that of the wild (Szokalski, Litchfield, and Foster 2012). But, De Rouck et al. (2005) found that tigers paced less when housed together. Galardi et al. (2021), noted strong pair interactions indicating a potential for preferred conspecifics within tigers, and Miller, Bettinger, and Mellen (2008) found that the inability to see or interact with conspecifics resulted in increased tiger pacing. Conversely, however, Bashaw et al. (2007) found that increased pacing occurred when tigers could see each other. It is, therefore, problematic to reference wild behaviour and use it to predict welfare in captive tigers (Koene 2013).

We scored singularly housed tigers as appropriate social grouping (see Supporting Information S2: Appendix 2), but we acknowledge that this is debatable and further research is needed for conclusive evidence. Our reason for scoring in this manner was also due to the limited enclosure size, variations in tiger age and sex and the likelihood of tiger groups being unrelated, factors that meant the tigers were not compatible in these smaller areas but would only be better living together in larger enclosures where they can choose to remain separate from each other (Galardi et al. 2021). The best social grouping will vary on individual tiger personalities, enclosure design and size and the way the facility is set up. In cases such as this, tiger history should be considered (Blache, Terlouw, and Maloney 2018). As such, more research is needed regarding the effects of social grouping in captive tigers.

Both positive behaviour and ARBs also affected welfare scores. Positive active behaviours, including scent marking, exploration, grooming, play and intraspecies interactions, were observed, as well as positive passive such as sleeping or resting behaviours. However, only four facilities received the highest score (Table 1), indicating a well‐rounded repertoire of positive behaviours in all living spaces (Supporting Information S2: Appendix 2). Nearly half the facilities had tigers displaying no positive behaviours. This does not mean that these tigers were showing negative or ARBs but that there was little to no interaction with the environment. In a quick assessment, passive behaviours are harder to differentiate between positive, relaxed behaviours such as sleeping or negative, bored or helpless behaviours, which may have contributed to the low scores here.

However, while it is possible that the tigers were simply performing natural passive behaviours – tigers are predominantly inactive for large portions of the day (Zhen‐sheng et al. 2002), particularly in the hotter parts of the day (Yang, Fingean, and Brown 2013) – we conclude that it is likely that these tigers were not stimulated due to the lack of enrichment and limited enclosure size and design, a likely conclusion given Environment had the lowest welfare score (Figure 1). The fact that ARBs were also observed in all facilities bar, three supports this argument. As with the scoring for positive behaviour, the lack of observed ARBs does not mean that positive behaviours were observed instead. Additionally, due to the snapshot nature of this assessment, it is possible that the time of day the tigers were observed may not have been carrying out positive behaviours, but this does not mean that they do not occur.

### 3.5 Mental State

Results showed that the number of tiger colour variants (white, snow and golden) and levels of entertainment interaction (cub feeding, tiger shows and tiger photos) had the strongest impact on Mental State scores. Facilities that had fewer colour variants and little to no entertainment activities had a better Mental State score than facilities with large numbers of colour variants and multiple tiger entertainment activities. The presence of colour variants impacted the welfare score immediately as the observation of a white, snow or golden tiger elicited a 0 score for inbreeding (Xu et al. 2013). Additionally, these colour variants were observed to have poorer BCSs (often obese) and more obvious signs of pain or injury, such as limping. White tigers, in particular, are very popular with tourists around the world, with facilities breeding large numbers to cater to this demand and increase revenue (WWF 2021). To achieve these colour variants on a consistent basis, facilities need to engage in heavy inbreeding (Xu et al. 2013), which subsequently ignores occurrences of health issues such as hip dysplasia, strabismus and scoliosis in these tigers (Allendorf et al. 2022; Bernays and Smith 1999). To further emphasis, the welfare issues surrounding tiger colour variants, the Association of Zoos and Aquariums (AZA) (2011) issued a ban on member facilities from breeding exotic colour variations across a range of species, including banning white tigers. Other zoo associations are yet to follow suit.

The data showed that a reduction in entertainment activities also improved the Mental State score. Entertainment activities involve tiger shows and interactions such as cub feeding and tiger photos (Cohen 2009, 2012; Schmidt‐Burbach, Ronfot, and Srisangiam 2015) where tigers interact with the public regularly. Regular human–animal interactions are known to be stressful to many species (Morgan and Tromborg 2007; Suárez, Recuerda, and Arias‐De‐Reyna 2017), negatively impacting welfare (Hosey and Melfi 2015). Entertainment activities such as these force the tigers to perform unnatural behaviours during shows as well as staying awake during the day in contrast to their natural behaviour (Szokalski, Litchfield, and Foster 2012), restrict movement through chaining for photos (Cohen 2013), preventing tigers from removing themselves from a stressor (Morgan and Tromborg 2007), and be exposed to increased noise levels, all of which have the potential to increase stress‐related behaviours (Broom 2014). Additionally, tigers used in such entertainment activities often undergo declawing practices, causing severe health problems down the line, thus increasing a lower score for pain and injury in our assessment. Cubs are removed at a young age from their parents (Cohen 2012; Schmidt‐Burbach, Ronfot, and Srisangiam 2015), potentially impacting the cub's mental and physical health (Ahola, Vapalahti, and Lohi 2017). As young cubs are lucrative in the tourism industry, overcrowding occurs due to speed breeding practices (WAP 2016) limiting access to resources (Schmidt‐Burbach, Ronfot, and Srisangiam 2015). It is likely that some facilities are overloading themselves and not factoring in space, funds and food. Additionally, the Thailand Animal Cruelty Prevention and Welfare Act (2014) does not have adequate legislative protection. Both these factors result in poor welfare across many areas.

Subsequently, the improvement in Mental State scores for tigers in facilities with no interaction activities is likely due to no interaction with unfamiliar humans. However, this does not mean that the overall environment was a good one to live in for these tigers. This is evidenced by the fact more than half of the facilities offered interactions of some kind indicating just how popular these are.

The number of tigers recorded at each facility was not an affecting factor on welfare. This may be a surprise as it could be posited that larger numbers of tigers could result in decreased welfare particularly as social grouping was a main factor impacting welfare. Sociality in tigers is not fully understood, and captivity may also affect this result. However, a number of the facilities that held large numbers of tigers offered relatively adequate welfare standards, thus bringing up the average. Some facilities with large numbers of tigers did have poor welfare, but in many cases, so did single‐housed tigers or facilities with very few tigers.

To fully determine the impact of these interactions on the welfare of captive tigers in Thailand, more data is needed to effectively analyze the welfare impact before, during and after an interaction. As such, a snapshot welfare assessment such as this is insufficient to effectively interpret the impact of human–animal interactions in this setting. However, the results do highlight welfare domains that are in clear need of improvement within tiger facilities and likely can be extrapolated for other species. With such tangible results, this research can be built in and improve legal recognitions to the existing legislation, specifically in areas such as physical environment. Results here can be used to clarify definitions within the Thai Animal Welfare Act (2014) and provide an improved animal welfare framework and national commitment to protecting endemic species.

## 4 Conclusion

The data showed nutrition provision scored the highest, followed by health, behavioural interaction with physical environment scoring the lowest. It is not surprising to find nutrition scoring highest as this pertains to basic food and water requirements which are covered in the Animal Cruelty Prevention and Welfare Act (2014). However, there are no zoo standards in place, resulting in minimum living space sizes being very common throughout Thailand. These do not meet the behavioural needs of tigers resulting in an array of negative behaviours observed. Additionally, the greater the number of colour variant tigers kept in a facility and the lower the entertainment score (i.e., more entertainment activities were available), the lower the mental domain score. This aligns with the poor health many colour variants suffer, as well as the belief that human–tiger interactions are potentially stressful.

To improve Thai facilities regarding tiger welfare, focus must be placed on improving current conditions. A move away from the entertainment model and breeding tiger colour variants needs to be encouraged. There is a need to educate facility management and staff encouraging welfare‐friendly experiences through the provision of enrichment programmes designed to increase living space complexity. Parallel to this, facilities need to be encouraged to move toward progressive, naturalistic and large spaces that can control ex‐situ breeding.

## Ethics Statement

This study was purely observational and was approved by the ‘For Tigers’ Trustee Board ahead of data collection. This research was conducted in accordance with the ARRIVE Guidelines where applicable, as well as the ethical standards required for publication in Zoo Biology.

## Conflicts of Interest

Tanya S. Erzinçlioğlu is the Founder and Director of ‘For Tigers’, a UK‐based charity (Charity Number: 1176840) working in Thailand to improve tiger facilities. The other authors declare no conflicts of interest.

## Supporting information


**Appendix 1.** Welfare Assessment score sheet.

**Appendix 2.** Guidelines and explanation for the Welfare Assessment score sheet.

## Data Availability

The data that support the findings of this study are available on request from the corresponding author. The data are not publicly available due to privacy or ethical restrictions.

## References

[zoo21866-bib-0001] Ahola, M. K. , K. Vapalahti , and H. Lohi . 2017. “Early Weaning Increases Aggression and Stereotypic Behaviour in Cats.” Scientific Reports 7: 10412. 10.1038/s41598-017-11173-5. 28871130 PMC5583233

[zoo21866-bib-0002] Allendorf, F. W. , W. C. Funk , S. N. Aitken , M. Byrne , and G. Luikart . 2022. Conservation and the Genomics of Populations, 3rd ed. Oxford: Oxford University Press. 10.1111/eva.13499 PMC9753819 36540641

[zoo21866-bib-0003] Altmann, J. 1974. “Observational Study of Behavior: Sampling Methods.” Behaviour 49, no. 3/4: 227–266. 4597405 10.1163/156853974x00534

[zoo21866-bib-0004] Animal Cruelty Prevention and Welfare Act . 2014. “Thailand Animal Anti Cruelty and Welfare” [Online]. Global Animal Law. https://www.globalanimallaw.org/database/national/thailand/.

[zoo21866-bib-0005] Association of Zoos and Aquariums (AZA) . 2011. *Welfare and Conservation Implications of Intentional Breeding for the Expression of Rare Recessive Alleles* [Online]. Association of Zoos & Aquariums. https://assets.speakcdn.com/assets/2332/aza_white_paper_inbreeding_for_rare_alleles_18_jan_2012.pdf.

[zoo21866-bib-0006] AZA Tiger Species Survival Plan . 2016. Tiger Care Manual. Silver Spring, MD: Association of Zoos and Aquariums.

[zoo21866-bib-0007] Bashaw, M. J. , A. S. Kelling , M. A. Bloomsmith , and T. L. Maple . 2007. “Environmental Effects on the Behavior of Zoo‐Housed Lions and Tigers, With a Case Study of the Effects of a Visual Barrier on Pacing.” Journal of Applied Animal Welfare Science 10, no. 2: 95–109. 17559318 10.1080/10888700701313116

[zoo21866-bib-0008] Bernays, M. , and R. Smith . 1999. “Convergent Strabismus in a White Bengal Tiger.” Australian Veterinary Journal 77, no. 3: 152–155. 10197239 10.1111/j.1751-0813.1999.tb11220.x

[zoo21866-bib-0009] Biolatti, C. , P. Modesto , D. Dezzutto , et al. 2016. “Behavioural Analysis of Captive Tigers (*Panthera tigris*): A Water Pool Makes the Difference.” Applied Animal Behaviour Science 174: 173–180.

[zoo21866-bib-0010] Blache, D. , C. Terlouw , and S. K. Maloney . 2018. “Physiology.” In Animal Welfare, 3rd ed., edited by M. C. Appleby , I. A. S. Olsson , and F. Calindo , 181–212. Wallingford, UK: CAB International.

[zoo21866-bib-0011] Breton, G. , and S. Barrot . 2014. “Influence of Enclosure Size on the Distances Covered and Paced by Captive Tigers (*Panthera tigris*).” Applied Animal Behaviour Science 154: 66–75.

[zoo21866-bib-0012] Broom, D. 2014. Sentience and Animal Welfare. Wallingford, UK: CAB International.

[zoo21866-bib-0013] Carlstead, K. , and D. Shepherdson . 1994. “Effects of Environmental Enrichment on Reproduction.” Zoo Biology 13: 447–458.

[zoo21866-bib-0014] Carr, N. 2016. “Ideal Animals and Animal Traits for Zoos; General Public Perspectives.” Tourism Management 57: 37–44.

[zoo21866-bib-0015] Clark, K. , T. Bailey , P. Rist , and A. Matthews . 2014. “Comparison of 3 Methods of Onychectomy.” Canadian Veterinary Journal = La revue veterinaire canadienne 55, no. 3: 255–262. 24587509 PMC3923482

[zoo21866-bib-0016] Cohen, E. 2009. “The Wild and the Humanized: Animals in Thai Tourism.” Anatolia 20, no. 1: 100–118.

[zoo21866-bib-0017] Cohen, E. 2012. “Tiger Tourism: From Shooting to Petting.” Tourism Recreation Research 37, no. 3: 193–204.

[zoo21866-bib-0018] Cohen, E. 2013. “‘Buddhist Compassion’ and ‘Animal Abuse’ in Thailand's Tiger Temple.” Society & Animals 21, no. 3: 266–283. 10.1163/15685306-12341283.

[zoo21866-bib-0019] Convention on International Trade in Endangered Species of Wild Fauna and Flora (CITES) . 2019. Facilities Which May Be of Concern Keeping Asian Big Cats (*Felidae* Spp.) in Captivity. Sri Lanka: Seventy‐First Meeting of the Standing Committee Colombo. https://cites.org/sites/default/files/eng/com/sc/71/E-SC71-19.pdf.

[zoo21866-bib-0020] Corrigan, A. , L. Ng , and M. Williamson . 2010. Investigation Into the Welfare Standards of Zoos in Malaysia. Singapore: ACRES.

[zoo21866-bib-0021] Croney, C. C. , C. L. Daigle , M. Hurt , and J. L. Stella . 2015. Effects of Flooring on Animal Health and Well‐Being: Implications for Kenneled Dogs , 1–4. Perdue Extension. https://extension.purdue.edu/extmedia/VA/VA‐4‐W.pdf?_ga=2.136128497.1140902069.1638396355‐24029755.1637129144.

[zoo21866-bib-0022] Damasceno, J. , G. Genaro , T. Quirke , S. McCarthy , S. McKeown , and R. O'Riordan . 2017. “The Effects of Intrinsic Enrichment on Captive Felids.” Zoo Biology 36: 186–192. 29165868 10.1002/zoo.21361

[zoo21866-bib-0023] De Rouck, M. , A. Kitchener , G. Law , and M. Nelissen . 2005. “A Comparative Study of the Influence of Social Housing Conditions on the Behaviour of Captive Tigers (*Panthera tigris*).” Animal Welfare 14: 229–238.

[zoo21866-bib-0024] Dierenfeld, E. S. , M. Bush , L. Phillips , and R. Montali . 1994. “Nutrition, Food Preparation and Feeding.” In Management and Conservation of Captive Tigers Panthera tigris, edited by R. Tilson , G. Brady , K. Traylor‐Holzer , and D. Armstrong , 47–52. Apple Valley, Minnesota: Minnesota Zoo.

[zoo21866-bib-0025] Dorloh, S. 2017. “The Protection of Animals in Thailand – An Insight Into Animal Protection Legislation.” International Journal of Humanities and Cultural Studies 4, no. 2: 58–63.

[zoo21866-bib-0802] Environmental Investigation Agency (EIA) . 2017. “Cultivating Demand: The Growing Threat of Tiger Farms” [Online]. EIA, November 2017. https://eia-international.org/wp‐content/uploads/Cultivating‐Demand‐The‐Growing‐Threat‐of‐Tiger‐Farms.pdf.

[zoo21866-bib-0026] Environmental Investigation Agency (EIA) . 2020. “On the Butcher's Hook” [Online]. *EIA*, March 2020. https://eia-international.org/wp-content/uploads/On-the-Butchers-Block-Tigers-Mekong-Report-2020-SCREEN-SINGLE-PAGES.pdf.

[zoo21866-bib-0027] Fazio, J. 2020. “Felid Body Condition Poster” [Online]. *Felid Tag*. https://nagonline.net/3877/body-condition-scoring/.

[zoo21866-bib-0028] Galardi, E. G. , M. Fabbroni , F. A. Rausa , R. Prziosi , J. E. Brereton , and G. Q. Pastorino . 2021. “An Investigation Into the Behaviour, Sociality and Enclosure Use of Group‐Housed Lions and Tigers.” Journal of Veterinary Medicine and Animal Science 4, no. 1: 1068.

[zoo21866-bib-0029] Ghosh, N. 2016. “Thailand Introduces New Laws to Tackle Animal Cruelty” [Online]. *Straits Times*, February 23, 2016. http://www.straitstimes.com/asia/thailand-introduces-new-laws-to-tackle-animal-cruelty.

[zoo21866-bib-0030] Godinez, A. M. , and E. J. Fernandez . 2019. “What Is the Zoo Experience? How Zoos Impact a Visitor's Behaviors, Perceptions, and Conservation Efforts.” Frontiers in Psychology 10: 1746. 10.3389/fpsyg.2019.01746. 31417469 PMC6682629

[zoo21866-bib-0032] Gray, J. 2017. Zoo Ethics: The Challenges of Compassionate Conservation. New York: Cornell University Press.

[zoo21866-bib-0033] Hosey, G. , and V. Melfi . 2015. “Are We Ignoring Neutral and Negative Human‐Animal Relationships in Zoos?” Zoo Biology 34, no. 1: 1–8. 25328013 10.1002/zoo.21182

[zoo21866-bib-0034] Isoux, C. 2016. “Is Thailand Serious About Curbing Trade in Tigers?” [Online]. *South China Morning Post*, September 9, 2014. http://www.scmp.com/magazines/post-magazine/long-reads/article/2017620/thailand-serious-about-curbing-trade-tigers.

[zoo21866-bib-0036] Koene, P. 2013. “Behavioral Ecology of Captive Species: Using Behavioral Adaptations to Assess and Enhance Welfare of Nonhuman Zoo Animals.” Journal of Applied Animal Welfare Science 16, no. 4: 360–380. 24079489 10.1080/10888705.2013.827917

[zoo21866-bib-0037] Lyons, J. , R. J. Young , and J. M. Deag . 1997. “The Effects of Physical Characteristics of the Environment and Feeding Regime on the Behavior of Captive Felids.” Zoo Biology 16: 71–83.

[zoo21866-bib-0038] McPhee, M. E. 2002. “Intact Carcasses as Enrichment for Large Felids: Effects on On‐ and Off‐Exhibit Behaviors.” Zoo Biology 21, no. 1: 37–47.

[zoo21866-bib-0039] Melfi, V. A. , W. McCormick , and A. Gibbs . 2004. “A Preliminary Assessment of How Zoo Visitors Evaluate Animal Welfare According to Enclosure Style and the Expression of Behavior.” Anthrozoös 17, no. 2: 98–108.

[zoo21866-bib-0040] Mellor, D. 2016. “Updating Animal Welfare Thinking: Moving Beyond the ‘Five Freedoms’ Towards ‘A Life Worth Living’.” Animals 6, no. 3: 1058–1072. 10.3390/ani6030021 PMC4810049 27102171

[zoo21866-bib-0041] Mellor, D. J. , N. J. Beausoleil , K. E. Littlewood , et al. 2020. “The 2020 Five Domains Model: Including Human‐Animal Interactions in Assessments of Animal Welfare.” Animals 10, no. 10: 1870. 10.3390/ani10101870. 33066335 PMC7602120

[zoo21866-bib-0042] Miller, L. , T. Bettinger , and J. Mellen . 2008. “The Reduction of Stereotypic Pacing in Tigers (*Panthera tigris*) by Obstructing the View of Neighbouring Individuals.” Animal Welfare 17, no. 3: 255–258.

[zoo21866-bib-0043] Mishra, A. K. , R. K. Mohapatra , S. P. Parida , and S. Mishra . 2021. Environmental Enrichment: A Prospective of Captive Felid Management. Chennai: Notion Press.

[zoo21866-bib-0044] Morgan, K. N. , and C. T. Tromborg . 2007. “Sources of Stress in Captivity.” Applied Animal Behaviour Science 102, no. 3: 262–302.

[zoo21866-bib-0045] Nyhus, P. J. , R. Tilson , and M. Hutchins . 2010. “Thirteen Thousand and Counting: How Growing Captive Tiger Populations Threaten Wild Tigers.” In Tigers of the World. The Science, Politics, and Conservation of Panthera tigris, 2nd ed., edited by R. Tilson and P. J. Nyhus , 223–238. London, UK: Elsevier.

[zoo21866-bib-0046] Olsson, I. A. S. , H. Wurbel , and J. A. Mench . 2018. “Behaviour.” In Animal Welfare, 3rd ed., edited by M. C. Appleby , I. A. S. Olsson , and F. Calindo , 160–180. Wallingford: CAB International.

[zoo21866-bib-0047] Reade, L. S. , and N. K. Waran . 1996. “The Modern Zoo: How Do People Perceive Zoo Animals?” Applied Animal Behaviour Science 47: 109–118.

[zoo21866-bib-0048] Ritzler, C. P. , K. E. Lukas , L. M. Bernstein‐Kurtycz , and D. C. Koester . 2021. “The Effects of Choice‐Based Design and Management on the Behavior and Space Use of Zoo‐Housed Amur Tigers (*Panthera tigris altaica*).” Journal of Applied Animal Welfare Science 26: 256–269. 10.1080/10888705.2021.1958684. 34353192

[zoo21866-bib-0049] Rose, P. E. , S. M. Nash , and L. M. Riley . 2017. “To Pace or Not to Pace? A Review of What Abnormal Repetitive Behavior Tells Us About Zoo Animal Management.” Journal of Veterinary Behavior 20: 11–21.

[zoo21866-bib-0050] Sanderson, E. W. , J. Forrest , C. Loucks , et al. 2010. “Setting Priorities for Tiger Conservation: 2005–2015.” In Tigers of the World: The Science, Politics, and Conservation of Panthera tigris, 2nd ed. , edited by R. Tilson and P. J. Nyhus . Amsterdam: Boston Elsevier/Academic Press.

[zoo21866-bib-0051] Schmidt‐Burbach, J. , D. Ronfot , and R. Srisangiam . 2015. “Asian Elephant (*Elephas maximus*), Pig‐Tailed Macaque (*Macaca nemestrina*) and Tiger (*Panthera tigris*) Populations at Tourism Venues in Thailand and Aspects of Their Welfare.” PLoS One 10, no. 9: e0139092. 10.1371/journal.pone.0139092. 26407173 PMC4583339

[zoo21866-bib-0052] Skibiel, A. L. , H. S. Trevino , and K. Naugher . 2007. “Comparison of Several Types of Enrichment for Captive Felids.” Zoo Biology 26: 371–381. 19360587 10.1002/zoo.20147

[zoo21866-bib-0053] Stryker, J. A. , J. L. Atkinson , R. D. Brown , et al. 2019. “Behavioral Repertoire Assessment of Bengal Tigers (*Panthera tigris*) With Focus on Thermoregulatory Behavior.” International Journal of Biometeorology 63: 1369–1379. 31309283 10.1007/s00484-019-01753-7

[zoo21866-bib-0054] Suárez, P. , P. Recuerda , and L. Arias‐De‐Reyna . 2017. “Behaviour and Welfare: The Visitor Effect in Captive Felids.” Animal Welfare 26, no. 1: 25–34.

[zoo21866-bib-0055] Sunquist, M. , and F. Sunquist . 2002. Wild Cats of the World. Chicago and London: University of Chicago Press.

[zoo21866-bib-0056] Szokalski, M. S. , C. A. Litchfield , and W. K. Foster . 2012. “Enrichment for Captive Tigers (*Panthera tigris*); Current Knowledge and Future Directions.” Applied Animal Behaviour Science 139, no. 1–2: 1–9.

[zoo21866-bib-0057] Tarou, L. R. , and M. J. Bashaw . 2007. “Maximizing the Effectiveness of Environmental Enrichment: Suggestions From the Experimental Analysis of Behavior.” Applied Animal Behaviour Science 102: 189–204.

[zoo21866-bib-0058] The Scottish Government . 2019. *Lions and Tigers – Dangerous Wild Animals: Species Guidance*. https://www.gov.scot/publications/dangerous-wild-animals-species-guidance/pages/lions-tigers/.

[zoo21866-bib-0059] Tilson, R. L. , and U. S. Seal . 1987. Tigers of the World: The Biology, Biopolitics, Management, and Conservation of an Endangered Species. Park Ridge, New Jersey: Noyes Publications.

[zoo21866-bib-0060] Veasey, J. S. 2020. “Can Zoos Ever Be Big Enough for Large Wild Animals? A Review Using an Expert Panel Assessment of the Psychological Priorities of the Amur Tiger (*Panthera tigris altaica*) as a Model Species.” Animals 10, no. 9: 1536. 10.3390/ani10091536. 32878205 PMC7552275

[zoo21866-bib-0801] Ward, S. J. , E. Williams , G. Groves , S. Marsh , and D. Morgan . 2020. “Using Zoo Welfare Assessments to Identify Common Issues in Developing Country Zoos.” Animals 10, no. 11: 2101. 10.3390/ani10112101. 33198237 PMC7696472

[zoo21866-bib-0061] Wipatayotin, A. 2020. “Wild Tiger Population Growing Fast” [Online]. *Bangkok Post*, July 24, 2020. https://www.bangkokpost.com/thailand/general/1956471/wild-tiger-population-growing-fast.

[zoo21866-bib-0062] World Animal Protection (WAP) . 2010. *Wildlife on a Tightrope: An Overview of Wild Animals in Entertainment in Thailand* [Online] (World Animal Protection). https://www.worldanimalprotection.org/sites/default/files/int_files/wildlife-on-a-tightrope-thailand.pdf.

[zoo21866-bib-0063] World Animal Protection (WAP) . 2016. *Tiger Selfies Exposed: A portrait of Thailand's Tiger Entertainment Industry* [Online] (World Animal Protection). https://www.worldanimalprotection.org/sites/default/files/int_files/tiger_selfies_exposed_a_portrait_of_thailands_tiger_entertainment_industry.pdf.

[zoo21866-bib-0064] World Wildlife Fund (WWF) . 2021. *The Truth About White Tigers* [Online] (WWF). https://www.worldwildlife.org/stories/the-truth-about-white-tigers.

[zoo21866-bib-0065] World Wildlife Fund (WWF) . 2022. *Tiger* [Online] (WWF). https://www.worldwildlife.org/species/tiger.

[zoo21866-bib-0066] Xu, X. , G. X. Dong , X. S. Hu , et al. 2013. “The Genetic Basis of White Tigers.” Current Biology 23, no. 11: 1031–1035. 23707431 10.1016/j.cub.2013.04.054

[zoo21866-bib-0067] Yang, T. , E. Fingean , and R. D. Brown . 2013. “Effects of Summer Microclimates on Behaviour of Lions and Tigers in Zoos.” International Journal of Biometeorology 57: 381–390. 22707238 10.1007/s00484-012-0562-6

[zoo21866-bib-0068] Zhen‐sheng, L. , L. Fend , T. Li‐Wei , and Z. Zioa‐yu . 2002. “Time Budget of Semi Free‐Ranging Amur Tigers (*Panthera tigris altaica*).” Zoological Research 5: 389–439.

