# Supplementary material for: An Analysis of Welfare Standards Within Tiger (Panthera tigris) Facilities in Thailand
Source: Zoo Biol. 2024 Sep 16;43(6):545–55. doi: 10.1002/zoo.21866 (PMC11624624; doi:10.1002/zoo.21866)
Supplement: Supplementary file 2 — Appendix 2. Guidelines and explanation for the Welfare Assessment score sheet. [file ZOO-43-545-s002.docx]

**Guidelines and Explanation for Welfare Assessment score sheet**

This welfare assessment only considers tiger welfare during the opening hours of the facilities and does not take into account back of house accomodation unless it can be seen by a tourist.

For all sections of this welfare assessment, the worst observed welfare is used as a basis for the score. In cases where there are conflicting results i.e. one tiger has water, but another doesn’t, or one tiger had access to a large enclosure while another was confined to a cage, the worst condition is chosen and scored.

Due to the subjective nature of some scoring such as pain, this guide provides details on how to score these areas.

There is no weighting involved when calculating the final scores with each section holding equal weight.

**Nutrition**

Water provision .

| 0 | If even one tiger is seen without access to water, a score of 0 must be given. |
| --- | --- |
| 1 | N/a |
| 2 | Water must be provided to all observed tigers in order to be awarded 2 |

Clean/running water

Following the previous assessment criteria (see water provided)

| 0 | if there was no water provided to a single tiger, the facility automatically scores 0. |
| --- | --- |
| 1 | N/a |
| 2 | All tigers must have access to clean, fresh and ideally running water. |

Species appropriate diet provided

| 0 | An incorrect diet is provided (i.e. cooked food or dog food), and/or not enough food or too much food is given. |
| --- | --- |
| 1 | The type of food is correct i.e. raw, but there is no variation (i.e. chicken only) and food is only given in bowls/floor. |
| 2 | Provide a tiger-specific diet including an appropriate carnivore supplement and variety within the diet including red meat, chicken and carcasses. |

Researchers considered whether the way the tiger is fed encourages natural behavior such as hunting or foraging (i.e. food enrichment). Facilities that provide food enrichment score higher. In some instances, it will be necessary for the researcher to question the staff regarding the diet as feeding is often performed after opening hours.

**Physical Environment**

Enclosure size

| 0 | A cage only, defined as a living space constructed of bars and mesh with a roof cover, results in a score of 0. Similarly, if there is an enclosure but it is still very small i.e. no larger than 4x4m, this also scores 0 as there is not enough space for the tiger to be able to run. |
| --- | --- |
| 1 | An enclosure allowing some running, but not enough space to reach a top speed/sprint is deemed to be medium and will score a 1. |
| 2 | A large enclosure allowing a full range of movement including jumping and the space to reach full speed will receive the highest score. |

Facilities offering a range of enclosure sizes will be scored on the smallest area.

Species-specific enclosure design:

| 0 | Facilities with no enclosure, where tigers are kept in cages only or even chained, receive the lowest score of 0. |
| --- | --- |
| 1 | Facilities that have a small enclosure (see enclosure size) or a larger cage that contains vegetation and an additional enrichment item will score a 1. |
| 2 | A medium/large enclosure (see enclosure size) that has a range of environmental additions such as a pond/platform/cave as well as a range of vegetation and substrate will score the highest. |

The length of time a tiger spends in the enclosure per day is not part of this assessment at this time. Future assessments may be modified to include this information. If known, researchers can add this information in the notes to be included at a future time.

Pond access:

| 0 | No pond in any areas results in a 0 score. If a pond structure is provided but does not contain water, a 0 is scored. |
| --- | --- |
| 1 | A small pond that allows the tiger to partially submerge in water recieves a 1. |
| 2 | A large pond or moat that enables full submersion and the ability to swim, while also having shallow resting areas receives the highest score of 2. |

Shelter access:

| 0 | Facilities that do not provide any type of shelter from the weather or a place for tigers to hide from view score 0. |
| --- | --- |
| 1 | Facilities that have some shelters i.e. a partial cave/over hang that allows shelter and the abilty for some tigers to hide will score a 1. |
| 2 | Facilities providing hiding places for all tigers under their care and full protection from the weather score highest. |

Cleanliness:

| 0 | Facilities that are unhygienic with faeces and rubbish are scored 0. |
| --- | --- |
| 1 | Facilities that are generally clean, have no rubbish with one or two areas that have some faeces score 1, any more than that suggests the enclosure hasn’t been cleaned for an extended period of time and should score 0. |
| 2 | Facilities that are clean in all areas score highest. |

Substrate variation:

| 0 | Facilities where tigers are housed on concrete or other unnatural substrates such as brick only, score 0. |
| --- | --- |
| 1 | Facilities where tigers are kept in concrete/brick-floored cages but have access to areas with other substrates such as grass, score 1. |
| 2 | Facilities with tigers kept on a range of natural substrates including dirt, sand, grass score 2. |

Environmental noise:

| 0 | Facilities where tigers are exposed to regular, consistent traffic either within the facility or from nearby roads, large crowds and /or with a consistent PA system or electronic noise, score 0. |
| --- | --- |
| 1 | Facilities that have some minor traffic such as the drive-through zoos and/or occasional electronic noise, score 1. |
| 2 | Facilities with only natural sounds and small visitor groups score the highest. |

Management/knowledge:

| 0 | Facilities that show no welfare understanding (i.e. tigers are overcrowded, have poor body condition and are kept on concrete), have no informative tiger-related signage and maintain a strong commercial interest score 0. |
| --- | --- |
| 1 | Facilities with improvements/rennovations to build new or improve existing living spaces recieve 1. |
| 2 | Facilities that show a good understanding of tiger welfare and captive tiger needs (i.e. large species-specific enclosure, no tourist interactions), score highest. |

Conversations with staff will help discern this score by discovering any specific knowledge of tigers in general and/or the specific tigers in their care.

**Health**

Signs of inbreeding:

| 0 | Any signs of inbreeding such as colour variants (i.e white, snow and golden), crossed eyes or deformities results in the lowest score |
| --- | --- |
| 1 | N/a |
| 2 | No observed signs of inbreeding. |

Some tigers may be inbred but without a stud book it is impossible to know and so observation only informs this score. Researchers can look for signs of inbreeding such as strabismus, hip dysplasia, and deformities to paws (club feet), face and back (short back syndrome).

Signs of injury:

| 0 | Large wounds, missing body parts (i.e. partial tails), hair loss, rubbing sores along cheekbones and face and/or large sores on other body parts particularly the elbow all result in the lowest score. Small wounds that appear infected should be scored 0. Very pink feet can have open wounds and should score 0. White tigers have pink pads which makes pad health observations harder. However, noticeable lesions or thinner skin on the pad should score 0. Even if only one tiger is seen with severe wounds/injuries, this results in the lowest score. |
| --- | --- |
| 1 | 1 is scored if the wounds or sores are small, scars or healed injuries and do not appear to hinder/cause pain. This can include scratches/minor injuries gained from general activity within the environment i.e. scratches from trees, toys or conspecifics. Any small injuries must appear to be treated (evidence of medical cleaning can be present), clean and not infected. Feet that have small patches of pink score 1. |
| 2 | A full score is only given if no injuries or hair loss of any kind is seen and if the paw pads are in good condition. |

If possible, researchers should take a look at the paws of the tiger. Orange tigers should have black paw pads. However, tigers kept on wet concrete for extended periods develop pink paw pads which are thinner and prone to greater wear and tear.

Signs of pain:

| 0 | Severe lameness (i.e. instantly observed) and heavy diarrhoea result in the lowest score of 0. Coughing/wheezing is given 0 no matter the level as this usually indicates a more serious medical condition. Other signs of pain in tigers include excessive licking at one place of the body, no grooming at all (unkempt, dirty or dull coat), hunched posture and stiffness in movement. Indicators such as hiding, vocalisations such as hissing (though this can also be from fear), dilated pupils (also from fear) and reduced appetite can also indicate pain, but are more likely to be recognisable only by keepers or by observers familiar with tiger behavior. Observation of any, or all, of these additional indicators results in a 0. Tigers with short-term/intense pain often show more outward signs, whereas chronic pain is often hidden, therefore any observations of the latter indicates a higher level of pain intensity and should score 0. |
| --- | --- |
| 1 | Slight minor limping that can only be determined after careful observation or faeces that are mainly solid with slight runniness results in a score of 1. Paw condition (see Signs of injury) can incur limping and pain and should be observed for this score as well. |
| 2 | No sign of pain noted |

Observing signs of pain, and differentiating between chronic and acute pain, can be difficult, so researchers should use the notes box in the Welfare Assessment sheet to write down any additional observations that they have used to inform their ‘Signs of pain’ score.

Body Condition score:

| 0 | Underweight (ribs clearly visible, protruding hipbones, high abdominal tuck, protruding cheekbones) or overweight (large layer of abdominal fat, no definition between rib cage and hips, fat layers visible across shoulder and chest) tigers are always scored 0. The lowest score is also given for poor coat condition such as missing fur, unkempt appearance, dirty (i.e. the tiger is not performing grooming behaviors – something that can indicate other issues). |
| --- | --- |
| 1 | ‘Adequate’ indicates acceptable coat condition possibly with some minor skin irritation. |
| 2 | Excellent body conditions should have visible ribs when tiger is moving, discernible hips, a primordial pouch that may be visible, but should look like loose skin, and a discernible slope from ribcage to hips. |

Health Care provided:

| 0 | Facilities that have no regular vet (often observable through the condition of the tigers), receive 0 |
| --- | --- |
| 1 | Facilities that do not have an onsite vet but get a vet in on occasion score 1 |
| 2 | Facilities with a permenant on-site vet score highest. |

This section usually requires the researcher to ask staff if there is a vet onsite.

**Behavior interactions**

Signs of stereotypy/ARB:

| 0 | Facilities score 0 if there are large numbers of tigers displaying intermediate levels of stereotypical or abnormal repetitive behaviors (ARB). The lowest score can also be given if only one or two tigers display very severe levels of stereotypy/ARB such as severe pacing/circling/route tracing, self-mutilation such as licking/chewing, bar rubbing, over-grooming, head bobbing etc. |
| --- | --- |
| 1 | A score of 1 is given for intermediate stereotypy/ARB where the smaller percentage of tigers within the facility are observered performing such behaviors, and to a more minor degree, i.e. they can be distracted from performing such behavior. |
| 2 | No stereotypy/ARB observed scores a 2, though this does not mean that positive behaviors were observed – the tiger could simply be asleep throughout the assessment. |

Response to non-threatening humans:

| 0 | Tigers that move away from the researcher or show a fearful/aggressive response when the researcher tries to engage with them are scored 0 |
| --- | --- |
| 1 | Human attempts at engagement that are met with disinterest, no movement to or from the researcher or no engagement, score 1. |
| 2 | Positive engagement with the researcher including moving toward the researcher, friendly greetings and curiousity score 2. |

Non-threatening humans are those that do not work with the tigers (no prior relationship) and are not holding objects that could be similar to those used to inflict pain (no sticks, whip-shaped objects etc).

Positive treatment by staff:

| 0 | Staff that act agressively or in other negative ways near or with the tiger score 0. |
| --- | --- |
| 1 | Keepers that are involved in hands-on or protected contact, but show disinterest or no engagement with the tiger score 1. No engagement can include the use of a guiding stick, pushing/holding tiger gently, but firmly in place, ignoring/looking away from the tiger, being distracted/excessive use of a phone, prioritising socialising with other staff members over paying attention to the tiger and no talking to/verbal encouragement toward the tiger. |
| 2 | Staff that are friendly/caring with the tigers directly or in the manner they discuss their work score 2. |

Keepers should be watched for a short time (5 minutes) to observe their behavior near the tigers and also in the way they talk about their work with the tigers – the latter because some facilities use protected contact only (where minimal physical contact is possible between keeper and tiger, through safety barriers such as a fence).

Signs of human applied injury:

| 0 | If any tiger is observed to be declawed/defanged the lowest score must be given, even if not all tigers undergo such practices. |
| --- | --- |
| 1 | N/a |
| 2 | If no human injury is observed, the highest score is awarded. |

Declawing can be seen due to the flatter, splayed shape of foot and is usually limited to the front feet. Researchers can ask staff. There is no intermediate here.

Positive behaviors observed:

| 0 | The lowest score is given if no tigers show any positive behaviors, though this does not mean they are showing negative, stereotypies or ARBs. |
| --- | --- |
| 1 | . A score of 1 is given if some tigers, but not all are showing positive behaviors, or if positive behaviors are shown in some, but not all areas. 1 is also given if only one or two positive behaviors are seen rather than a full repertoire. |
| 2 | To receive the highest score, tigers must be observed in all observable living spaces, with all performing positive/natural tiger behaviors. This can include scent marking, playing, exploring, foraging, positive social interactions with con-specifics as well as positive passive behaviors such as relaxing, sitting in ponds, grooming, sleeping. |

Positive behaviors were only scored for observable tigers. It is therefore likely that facilities would have scored lower here had all tigers been observed in all areas of the facility. Additionally, sleeping is considered positive as it is a natural behavior during the day. However, negative behavior such as learned helplessnes, lethargy and boredom, all potential negative welfare indicators could be mistaken for sleeping in this observational method.

Staff used physical force:

| 0 | The lowest score is given if any staff member is seen handling a tiger roughly in anyway. Even if sticks are used without hitting (some staff hold a stick to guide the tiger into position for a photo or use it to push the tigers head), this is scored as zero as on occasion these have been observed being used in a forceful manner or to intimidate. Facilities with circus-style tiger shows will score 0 due to the aggressive training. |
| --- | --- |
| 1 | A score of 1 is given if the facility is hands-on (allows interaction between tigers and keepers and tourists), but no sticks were used, no shows of force were witnessed and/or keepers used reward-based handling. |
| 2 | Facilities that use protected or no contact score 2. |

Enrichment provided:

| 0 | The lowest score is given if there is no enrichment provided. |
| --- | --- |
| 1 | Limited enrichment can include a single additional item such as a tyre/ball and recieve a score of 1. |
| 2 | The highest score is given if there is a range of different enrichment items available within the observable living space or if conversations with staff reveal there is an enrichment program in place. |

Items such as a platform/cave/pond in the cage/enclosure are not considered enrichment, but as environmental basics. Enrichment should include additional items within the enclosure to stimulate natural behaviors such as logs, hanging toys, balls, tyres and so on. It can include a feeding enrichment program/show.

Space; social grouping/interaction:

| 0 | Facilities where 2 or more tigers are kept in areas that are not appropriate for the number of tiger, for example where 2 or more tigers are held in a 4x4m cage or 15 tigers are in one enclosure, are scored 0. |
| --- | --- |
| 1 | Facilities that have some areas where the tigers are given more space, are not over crowded and are afforded appropriate social grouping, though may still have some tigers in inappropriate groupings are scored 1. |
| 2 | Facilities that hold 1 to 3 tigers in areas that are large enough to allow the tigers to remove themselves from any intra-species interaction are scored a 2. |

**Additional**

Entertainment level:

| 0 | Facilities that have a circus-style tiger show as well as photos and interaction opportunities will score 0. |
| --- | --- |
| 1 | Facilities that have no show, but still engage in interactions such as cub feeding or tiger photos score 1. |
| 2 | Facilities with no shows and no interactions score the highest. |
